# Anti-Müllerian hormone, testicular descent and cryptorchidism

**DOI:** 10.3389/fendo.2024.1361032

**Published:** 2024-03-04

**Authors:** Rodolfo A. Rey, Romina P. Grinspon

**Affiliations:** ^1^ Centro de Investigaciones Endocrinológicas “Dr. César Bergadá” (CEDIE), CONICET – FEI – División de Endocrinología, Hospital de Niños Ricardo Gutiérrez, Buenos Aires, Argentina; ^2^ Universidad de Buenos Aires, Facultad de Medicina, Departamento de Biología Celular, Histología, Embriología y Genética, Buenos Aires, Argentina; ^3^ Instituto de Investigaciones Biomédicas, Santa Fe, Argentina

**Keywords:** androgens, cryptorchidism, AMH, anti-Müllerian hormone, testis, testicular descent

## Abstract

Anti-Müllerian hormone (AMH) is a Sertoli cell-secreted glycoprotein involved in male fetal sex differentiation: it provokes the regression of Müllerian ducts, which otherwise give rise to the Fallopian tubes, the uterus and the upper part of the vagina. In the first trimester of fetal life, AMH is expressed independently of gonadotropins, whereas from the second trimester onwards AMH testicular production is stimulated by FSH and oestrogens; at puberty, AMH expression is inhibited by androgens. AMH has also been suggested to participate in testicular descent during fetal life, but its role remains unclear. Serum AMH is a well-recognized biomarker of testicular function from birth to the first stages of puberty. Especially in boys with nonpalpable gonads, serum AMH is the most useful marker of the existence of testicular tissue. In boys with cryptorchidism, serum AMH levels reflect the mass of functional Sertoli cells: they are lower in patients with bilateral than in those with unilateral cryptorchidism. Interestingly, serum AMH increases after testis relocation to the scrotum, suggesting that the ectopic position result in testicular dysfunction, which may be at least partially reversible. In boys with cryptorchidism associated with micropenis, low AMH and FSH are indicative of central hypogonadism, and serum AMH is a good marker of effective FSH treatment. In patients with cryptorchidism in the context of disorders of sex development, low serum AMH is suggestive of gonadal dysgenesis, whereas normal or high AMH is found in patients with isolated androgen synthesis defects or with androgen insensitivity. In syndromic disorders, assessment of serum AMH has shown that Sertoli cell function is preserved in boys with Klinefelter syndrome until mid-puberty, while it is affected in patients with Noonan, Prader-Willi or Down syndromes.

## 1 Introduction

**Figure 1:**
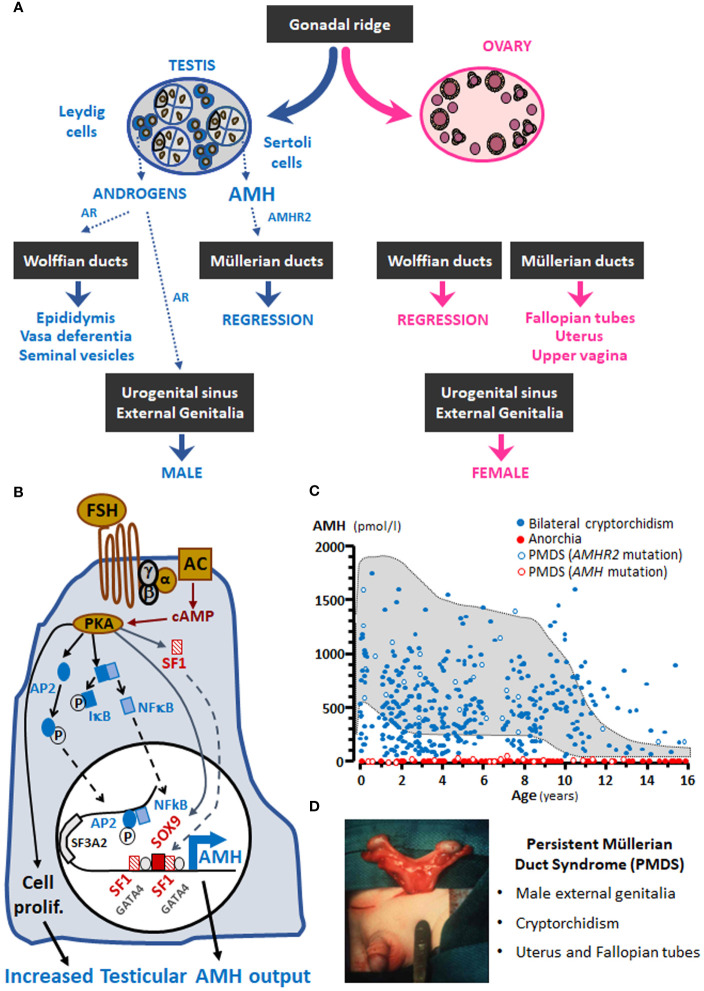
**(A)** AMH involvement in male fetal sex differentiation. **(B)** Regulation of testicular AMH production by FSH. **(C)** Serum AMH levels in boys with nonpalpable gonads; the shaded area represents normal levels. **(D)** PMDS: persistent Müllerian duct syndrome. α, β and γ, subunits of the Gs protein involved in FSH receptor signalling; AC, adenylyl cyclase; AP2, GATA4, IκB, NFκB, SF1 and SOX9, transcription factors involved in AMH expression regulation; AR, androgen receptor; cAMP, cyclic adenosine monophosphate; P, phosphorylated proteins; PKA, protein kinase A; prolif, proliferation. Modified with permission from: Rey and Grinspon (2) ^©^ 2010 Elsevier Ltd. (part A), Lasala et al. (3) ^©^ 2011 American Physiological Society (part B), and Josso et al. (4) ^©^ 2013 Hindawi Publishing Corporation (part C). Data in part C were obtained from Grinspon et al. (5) and Grinspon et al. (6).

The existence of a second testicular factor, in addition to testosterone, involved in fetal male sex differentiation was first suggested by the pioneering work carried out by the French scientist Alfred Jost in the 1940’s and 1950’s (1, 2). He called this factor “the Müllerian inhibitor” because it provoked the regression of the Müllerian ducts, the anlagen of the Fallopian tubes, the uterus and the upper portion of the vagina (**Figure 1A**). One of his trainees, the Parisian pediatrician Nathalie Josso, subsequently led the group that demonstrated that the “Müllerian inhibitor” −also called Müllerian inhibiting substance (MIS) or factor (MIF)− was a glycoprotein (7) secreted by the immature Sertoli cell (8), and named it anti-Müllerian hormone (AMH) (9).

### 1.1 AMH: the protein and the gene

AMH is a 560-aminoacid glycoprotein that contains a 24-aminoacid signal sequence. It is synthesized by the fetal Sertoli cell as a precursor homodimer of approximately 140 kDa, composed of a 110-kDa N-terminal pro-region and a 25-kDa C-terminal region. After post-translational modifications due to proteolysis (10), the N-terminal and C-terminal dimers stay associated forming a biologically active non-covalent complex (11, 12). When AMH binds to its receptor through its C-terminal domain, the N-terminal pro-region is displaced from the non-covalent complex (13). A cysteine knot motif in the C-terminal region of AMH and its post-translational processing are typical of the transforming growth factor beta (TGFβ) superfamily, to which AMH belongs (14).

The human *AMH* gene is approximately 2.75-kb long and consists of 5 exons (14). It maps to the short arm of chromosome 19 at position 13.3 (15), between *SF3A2*, encoding splicing factor 3A subunit 2 [also known as *SAP62* (16)] and *JSRP1*, coding for the junctional sarcoplasmic reticulum protein 1. Interestingly, both genes are less than 400 bp distant from *AMH*. The major transcription initiation site in the male gonad is 10 bp upstream of the ATG codon (17), the biologically active C-terminal domain is encoded by the 3’ end of the 5^th^ exon, and the polyadenylation signal is 90 nucleotides downstream of the TGA codon (14). A functional initiator (Inr) element in the human *AMH* gene is specifically recognized by transcription factor TFII-I (18). The proximal promoter has binding sites for SOX9, the main transactivating factor in the fetal Sertoli cell (3, 19), and for SF1, which is also a major regulator of AMH expression in humans (20) and rodents (3, 21–23). Response elements for GATA factors (22, 24, 25), SP1 (17) and AP1 (3) have also been found in the proximal 500 bp of the human *AMH* promoter (**Figure 1B**). Initially, the finding of *SF3A2*/*SAP62* less than 800 bp upstream of the ATG codon in the human *AMH* gene and less than 500 bp upstream in the mouse gene (16) abridged the attention to the proximal promoter. However, *in vivo* experimental studies in mice showed that more distant sequences are necessary for the maintenance of *Amh* expression after birth (26). Subsequent studies identified the physiological relevance of binding sites for AP2, NFκB (27) and the oestrogen receptor α (ERα) (28), present more than 1700 bp upstream of the ATG codon.

### 1.2 AMH expression

The testes are by far the most important source of AMH. The ovaries also produce AMH, though in much lesser amounts. In agonadal individuals, e.g. pure gonadal dysgenesis, bilateral anorchia, complete primary ovarian failure or post-castration, AMH is undetectable in serum (**Figure 1C**). Very tiny quantities of AMH have been detected in other organs, as discussed below.

#### 1.2.1 AMH expression in the testis and its regulation

AMH activity and its best known function are related to its testicular origin (1). AMH begins to be expressed in the fetal male gonad as soon as Sertoli cells differentiate, i.e. in the 6^th^ embryonic week (8 weeks of amenorrhea) in humans (29). Thus, AMH is one of the earliest markers of Sertoli cell differentiation and function. AMH protein expression is limited to the rough endoplasmic reticulum and the Golgi apparatus of Sertoli cells (30, 31). Although its best characterized action, provoking the regression of Müllerian ducts, is completed by the 10^th^ fetal week (32), AMH continues to be secreted by the testis throughout fetal and postnatal life.

Due to its time-restricted and sex-specific action during early fetal life, AMH synthesis and secretion is tightly regulated. The initiation of AMH expression in fetal Sertoli cells is independent of gonadotropins. The SRY-family transcription factor SOX9 triggers *AMH* gene transcription after binding to a specific response element present in the proximal promoter (19, 33, 34), 151 bp upstream of translation initiation site ATG, according to the most recent consensus nomenclature (35). Subsequently, SF1 upregulates AMH expression (21, 34) after binding to specific response elements at -102 and -228 of the human *AMH* promoter (20). Therefore, Müllerian duct regression occurs without any need for gonadotropin regulation of testicular function.

Sertoli cells continue to express high amounts of AMH during fetal life, infancy and childhood. FSH plays a role in the increase of AMH production by the testes, through two mechanisms (**Figure 1B**): it induces Sertoli cell proliferation and upregulates *AMH* gene expression (27, 36). FSH action is transduced by the classical G protein-coupled FSH receptor pathway involving the G_s_α subunit (37) and cyclic AMP (27, 38). Three downstream kinase-mediated mechanisms are involved in AMH transcriptional upregulation through proximal promoter sequences: PKA increases Sertoli cell expression of SOX9, SF1 and GATA4 as well as nuclear translocation of SF1; PI3K/PKB action increases the effects of SF1 and GATA4; and MEK1/2 and p38 MAPK enhance GATA4-mediated *AMH* upregulation (3). SF1 cooperates with GATA4 (39) and WT1 (40) to upregulate *AMH* transcription, while DAX1 counteracts the SF1/GATA4 action on AMH expression (41). On the other hand, PKA also boosts distal *AMH* promoter activity mediated by transcription factors AP2 and NFκB (27).

Finally, FSH induces aromatization of androgens to oestrogens, which upregulate *AMH* transcription involving ERα binding to distal AMH promoter sequences and, to a lesser extent, the G-coupled oestrogen receptor GPER, also called GPER1 or GPR30 (28).

During pubertal development, Sertoli cells undergo maturation, which is characterized by their proliferation arrest, a progressive decrease in AMH expression (42) and a switch of AMH directional secretion from the basal compartment, driving AMH to blood vessels in the interstitial tissue, to the adluminal compartment, directing AMH to the seminal fluid (43). Androgens are the major regulators of Sertoli cell maturation (44) and of AMH downregulation. Indeed, in *Tfm* mice with a natural androgen receptor (AR) defect (36) and in genetically modified mice lacking the AR in Sertoli cells (45), AMH expression persists high at pubertal age. Testosterone and dihydrotestosterone (DHT) downregulate the activity of the human *AMH* promoter despite the absence of canonical androgen response elements (ARE). The inhibitory effect of androgens on *AMH* expression is mediated either through blockage by interaction, i.e. an interaction between the AR and SF1, or through blockage by competition due to a direct binding of the AR on SF1 response elements in the proximal *AMH* promoter (23). The inhibitory effect of androgens prevails over the stimulating effect of FSH and oestrogens during pubertal maturation and adulthood (**Figure 1C**). The downregulation of testicular AMH production is coincident with other androgen-dependent processes, such as the establishment of the blood-testis barrier and the onset of adult spermatogenesis (36, 46, 47). Although intratesticular testosterone concentrations are high in the fetus and neonate, AMH is not downregulated owing to the lack of expression of the AR in Sertoli cells in those periods of life (48–50). In the adult male, AMH levels are 10- to 20-fold lower as compared to childhood (51), yet 2-fold higher than in females (52).

#### 1.2.2 AMH expression in other organs

In the ovary, AMH begins to be expressed in the 25^th^ fetal week (53), when Müllerian ducts are no longer sensitive to its action (32, 54). In humans, like shown in other mammals in the 1980´s (55, 56), AMH is produced mainly by granulosa cells of primary and small antral follicles, and decreases in large follicles; no AMH expression is seen in the corpus luteum, corpus albicans or atretic follicles (57, 58). As compared to the testis, ovarian AMH secretion is much lower and more stable throughout life, with a moderate peak during puberty or early adulthood and a decrease from the age of 25-30 years until menopause, when it becomes undetectable in serum (52, 59–61). The regulation of AMH expression in the ovary has received less attention than in the testis. FOXL2 and WNT4 are believed to trigger AMH expression in granulosa cells, with SF1 and GATA involved as upregulators. FSH and cyclic AMP, as well as members of the BMP family, have been shown to enhance AMH production in the ovary, whereas the effects of oestrogens and androgens remain unclear, probably depending on follicular stage [for review, see ref. (62)].

AMH mRNA has been found in other organs, such as neurons and gonadotrophs, using ultrasensitive techniques, which has prompted the hypothesis that AMH has other functions in the central nervous system (62–64). The fact that AMH cannot be detected in circulation in individuals lacking gonadal tissue and that patients with biallelic pathogenic variants in *AMH* or its specific receptor *AMHR2* only show the persistence of the uterus, with no neurologic symptoms (35) challenge these new findings or, at least, indicate that the proposed actions for AMH beyond fetal development are redundantly assured by other factors.

### 1.3 AMH action in target tissues

In concordance with members of the TGFβ family, AMH signals through two membrane-bound receptors with serine-threonine kinase activity. AMH must be cleaved −although the dissociation of the N-terminal and C-terminal fragments is not absolutely necessary− to bind to the specific AMH type 2 receptor (AMHR2). Subsequently, a nonspecific type 1 receptor is recruited by AMHR2 to transduce its signal (65). The type 1 receptor used varies according to the target tissue: in the mesenchymal cells that surround the Müllerian duct epithelium, AMHR2 recruits type 1 receptors ACVR1 and BMPR1A to phosphorylate intracellular proteins SMAD1/5/8 (62). A few target genes have been identified in rodents, including *Osx*, *Mmp2* and *Wif1* (66). AMH induces apoptosis and epithelial-mesenchymal transformation, finally leading to Müllerian duct regression (67). In the female fetus, lacking AMH expression during the critical period, and in male fetuses with abolished AMH production, owing to *AMH* gene mutations, or action, due to *AMHR2* mutations, Müllerian ducts are maintained and differentiate into the Fallopian tubes, the uterus and the upper third of the vagina. This condition, replicated in male mice with *Amh* (68) or *Amhr2* (69) gene knockouts, is known as the persistent Müllerian duct syndrome (PMDS, **Figure 1D**) (65). Müllerian derivatives also persist in individuals with dysgenetic gonads leading to insufficient testicular hormone production and resulting in disorders of sex development (DSD) (70).

## 2 AMH and testicular descent during fetal life

**Figure 2:**
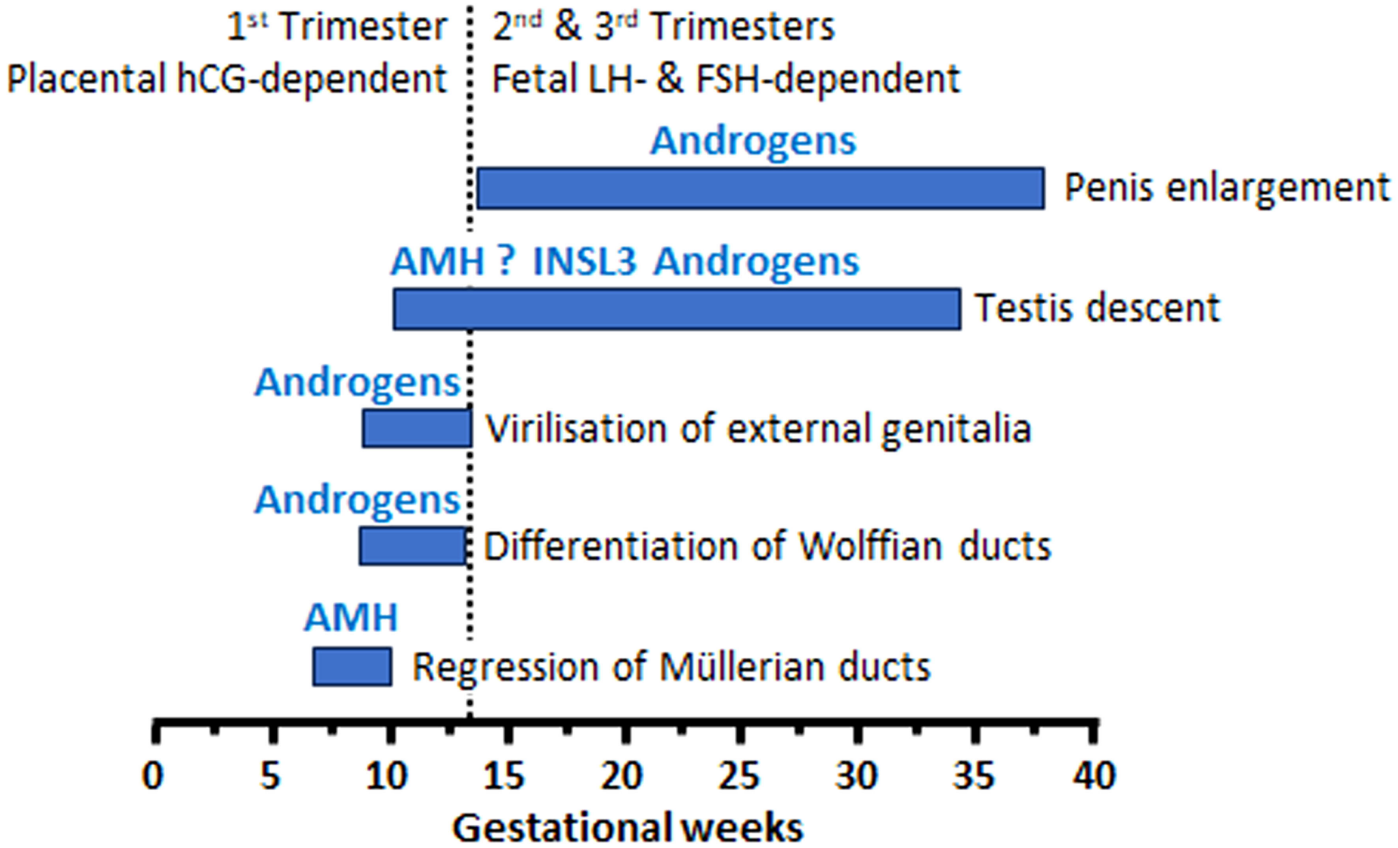
Hormones involved in male sex differentiation, including testicular descent during fetal life.

The biphasic model of fetal testicular descent is the most accepted proposal to explain how the testes descend from their original intra-abdominal position, when they differentiate from the urogenital ridge in the 7^th^ week, to the scrotum a few weeks before birth (71–74). The three hormones produced by the differentiating testis, AMH, androgens and insulin-like peptide 3 (INSL3), have been suggested to promote testis descent (**Figure 2**). The fetal testis is attached to the abdominal wall by the cranial suspensory ligament at its upper pole and the gubernaculum at its lower pole together with the epididymis. The initial or transabdominal phase of testicular descent is characterized by the regression of the cranial ligament and the thickening of the gubernaculum attaching to the inguinal region. In the second or inguino-scrotal phase, the testis descends along the inguinal canal into the scrotum.

INSL3 is the main testicular factor that controls the enlargement of the gubernaculum (73, 74), whereas androgens play some role in the gubernaculum swelling reaction. Androgens also provoke the regression of the cranial suspensory ligament (71). Both INSL3 and androgens are secreted by Leydig cells under the stimulus of human chorionic gonadotrophin (hCG) in the first 10-20 weeks of fetal life and LH in the second part of gestation.

The role of AMH in testis descent remains controversial. Until the 1990´s AMH was proposed as a candidate for the swelling reaction of the gubernaculum (75), but INSL3 was later recognized as the main responsible (71). Further observations in mice provided experimental evidence against an action for AMH on the gubernaculum (68, 76). However, *in vitro* studies showed that AMH might enhance the effect of INSL3 on the gubernaculum (77). Furthermore, in patients with PMDS due to *AMH* or *AMHR2* inactivating mutations, testicular descent does not occur normally. There are 3 presentations of cryptorchidism in patients with PMDS (35, 71): in the first one, both testes remain in intraabdominal position attached to the uterus and Fallopian tubes (similar to the normal ovarian position); in the second presentation, one testis is scrotal tracking the homolateral Fallopian tube and the contralateral testis remains intraabdominal (this is known as “hernia uteri inguinalis”); finally, the third situation is characterized by the presence of both testes, Fallopian tubes and uterus in one hemi-scrotum (known as “transverse testicular ectopia”). In all conditions the gubernaculum is long, indicating a lack of the swelling reaction, which could be explained by AMH signaling failure (71). The unusually long gubernaculum allows abnormal mobility of the testes, which are at increased risk of torsion (78). Alternatively, cryptorchidism in patients with PMDS could be explained by the retention of Müllerian derivatives which prevent testicular descent owing to a mechanical obstruction (35). In summary, there is no clear evidence that AMH is involved in testicular descent: although clinical observations in patients with PMDS seem to suggest a role for AMH in the INSL3-mediated swelling of the gubernaculum, experimental evidence could not be obtained from rodent models, probably due to species differences as regard the physiology of testicular descent between rodents and humans (71). In fact, in the human the gubernacular cord shortens in mid gestation explaining in part that testicular descent is usually complete by birth, whereas rodents have a long gubernacular cord that enables the testis inside the abdomen or the inguinal canal until puberty. Therefore, AMH may have a more impactful effect on INSL3-mediated control of the gubernacular swelling reaction in humans than in rodents (71). The demonstration of a timely expression of AMHR2 and its downstream signaling pathway in the gubernaculum is essential to support the hypothesis of AMH involvement in testicular descent during fetal life.

## 3 AMH as a biomarker of testicular function

Beyond its physiological role in Müllerian duct regression during the first trimester of fetal life and its potential roles in the physiology of the ovary and the hypothalamic-gonadotroph axis, AMH has proved to be a useful biomarker of testicular function and, especially, of its most conspicuous cell population, the immature Sertoli cell, in pediatric ages (4, 79–81).

The circulating AMH levels are commensurate with the mass of functional Sertoli cells in the fetus (29), newborns (82), infants and children (51, 83). In males of pubertal age, serum AMH is indicative of the maturation status of Sertoli cells, reflecting testosterone concentration and action within the testis (84, 85). This is especially useful in the early stages of pubertal development, i.e. Tanner stages 2 and 3 (86), when circulating testosterone may still be very low or undetectable (51, 87, 88).

Measurement of serum AMH is informative of the amount of functional testicular tissue in patients with DSD (89). Serum AMH is undetectable, indicating a complete absence of functional testicular tissue, in patients with complete gonadal dysgenesis, while it is below the male range −and probably above the female range− in patients with partial testicular dysgenesis. Conversely, serum AMH is in the male range or above in patients with isolated steroidogenic defects or androgen insensitivity. In 46,XX virilized patients, serum AMH is within the female range, indicating absence of testicular tissue, when the etiology is aromatase deficiency, androgen-secreting tumors or congenital adrenal hyperplasia. Conversely, it is above the female range in the case of 46,XX ovotesticular or testicular DSD (90).

In normally virilized 46,XY boys of prepubertal age with enlarged testes, low serum AMH is suggestive of precocious puberty, either central (87, 91) or peripheral (87), and its increase after treatment is indicative of successful reduction of intratesticular hormone concentration (87, 91). On the other hand, normal or high AMH levels rule out precocious maturation and suggest prepubertal macroorchidism in boys under 9 years with testicular volume ≥4 ml. High AMH may also reflect the existence of hypoestrogenic states (28), excessive FSH downstream signaling (37) or Sertoli cell tumors (92). The usefulness of serum AMH as a biomarker in patients with conditions that may present with cryptorchidism will be addressed in detail below.

## 4 AMH in boys with cryptorchidism

Cryptorchidism is the clinical sign resulting from an impaired testicular descent during fetal life (93), or from the re-ascent occurring later in life, when the cremasteric reflex is established (94). Cryptorchidism may be the consequence of a primary testicular dysfunction, of a central disorder affecting the GnRH-gonadotroph axis, or of an anatomic malformation independent of any endocrine disorder (**Table 1**) (72). Whichever the case may be, cryptorchidism is a frequent complaint in infants and children brought to the pediatrician at an age when the LH-Leydig cell axis is quiescent. Therefore, the assessment of the existence and the mass of functional testicular tissue relies mainly on the assessment of the Sertoli cell population. The clinical utility of measuring serum AMH levels in boys with cryptorchidism will be discussed.

**Table 1:** Pathophysiology of congenital cryptorchidism.

Primary hypogonadism	Whole testicular dysfunction	Testicular dysgenesis in 46,XY DSD Testicular dysgenesis in sex chromosome DSD with a Y chromosome (45,X/46,XY, 46,XX/47,XXY, etc.) 46,XX ovotesticular DSD 46,XX testicular DSD 46,XY testicular regression syndrome Klinefelter syndrome Noonan syndrome Trisomy 21 Prader-Willi syndrome Other rarer syndromes
	Leydig cell-specific dysfunction	Leydig cell hypoplasia/aplasia Steroidogenic defects INSL3 defects
Central hypogonadism	Whole testicular dysfunction	Combined pituitary hormone deficiency Isolated gonadotropin deficiency with hyposmia/anosmia (Kallmann syndrome) Isolated gonadotropin deficiency without hyposmia/anosmia
	Leydig cell-specific dysfunction	LHβ-subunit deficiency
Target organ defects	DHT deficiency	5α-reductase type 2 deficiency
	Androgen insensitivity syndrome	Androgen receptor defects
Anatomic defects	Abdominal wall defects	Prune belly syndrome Cloacal malformations
Ill-known mechanisms	PMDS	*AMH* mutations *AMHR2* mutations
	Idiopathic isolated cryptorchidism	

DHT, dihydrotestosterone; DSD, disorder of sex development; PMDS, persistent Müllerian duct syndrome.

### 4.1 Boys with nonpalpable gonads: cryptorchidism *vs* anorchia

**Figure 3:**
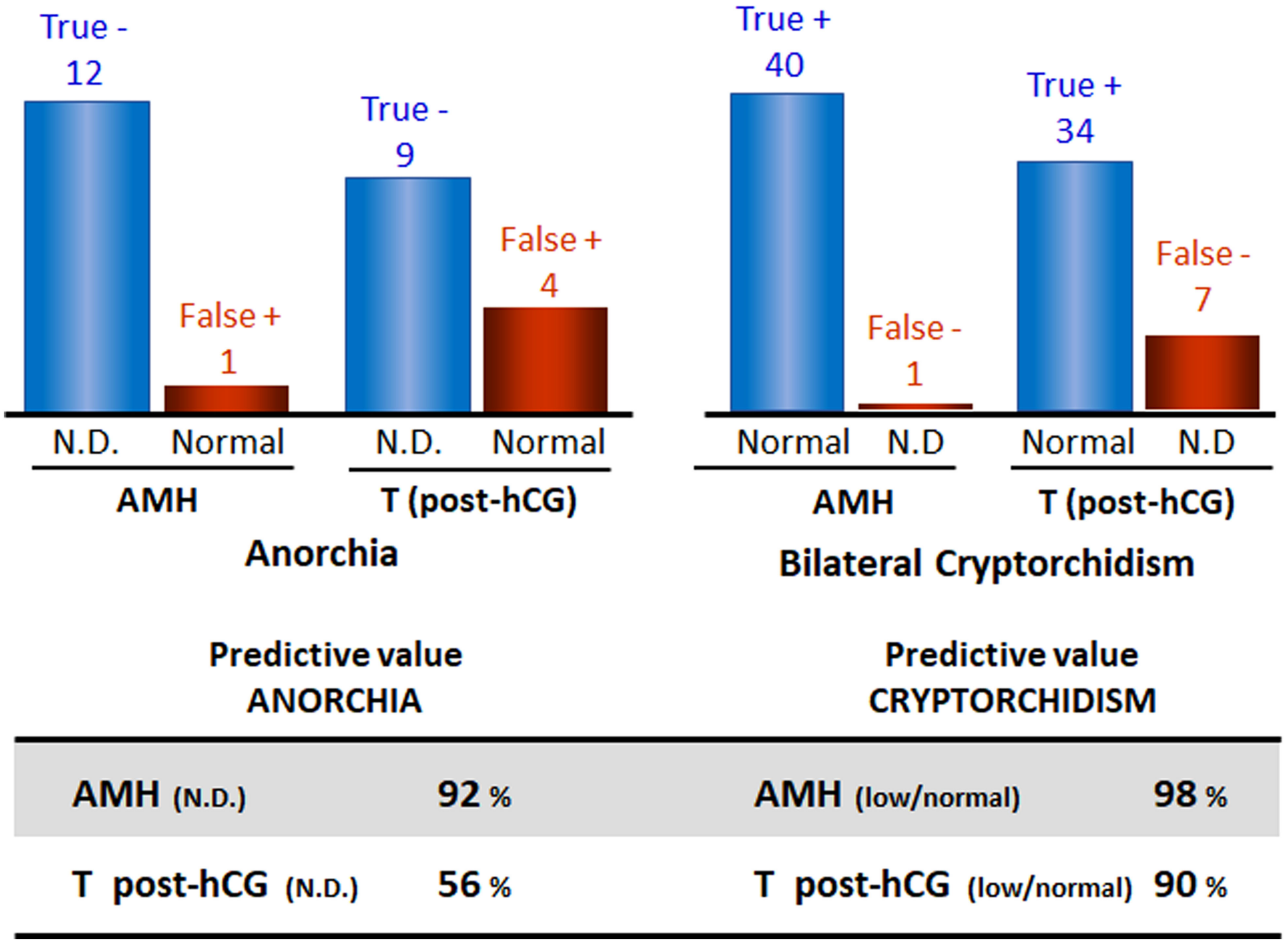
Predictive values of serum AMH and testosterone (T) post-hCG in boys with nonpalpable gonads for the existence of functional testicular tissue. Data from: Lee et al. (96). N.D., non-detectable.

When the gonads are not in the scrotum and cannot be detected in the inguinal regions by palpation or by ultrasonography, the first step in the diagnostic process is to establish the existence of functional testicular tissue. Basal serum gonadotropins and testosterone do not prove useful: testosterone is usually undetectable in serum after the age of 3-6 months (51, 95) and gonadotrophin levels may be normal even in the absence of testicular tissue (5). Testosterone levels measured after hCG stimulation were used in the past searching for the existence of functional testicular tissue; however, it is no longer a choice except when there is a specific interest in assessing Leydig cell function. Direct markers of the existence of prepubertal testicular tissue, such as AMH or inhibin B, have become the first choice. Indeed, basal serum AMH proves to be more informative than testosterone post-hCG both in boys with abdominal testes and in boys with anorchia (96). The predictive value of detectable serum AMH for the presence of testes was 98% and that of undetectable serum AMH for anorchia was 92% (**Figure 3**); the only false negative result was observed in a boy with PMDS due to an *AMH* mutation who had testes but serum AMH was undetectable.

Furthermore, serum AMH assessment has better predictive values than imaging studies for abdominal testes (97, 98) and may be more sensitive than surgical procedures for the identification of the existence of testicular tissue (99).

### 4.2 Assessment of testicular function in boys with isolated cryptorchidism

For decades, the treatment of cryptorchidism has focused on bringing the testes to the scrotum in an adequate and timely manner, as if placing the gonads in their proper position solved everything, without much attention having been paid to their functional status. However, it seems clear that assessing testicular function at the time of diagnosis can help to understand the pathophysiology of cryptorchidism in each patient and its potential fertility prognosis, as well as to appraise the effect of treatment.

Serum AMH levels are commensurate with the amount of functional testicular tissue. Most studies have found that serum AMH is overall lower in boys with cryptorchidism than in the general population (100, 101). However, AMH levels are not systematically decreased in all boys with cryptorchidism, and a few studies could not detect a significant difference as compared to control boys (102, 103). In boys with cryptorchidism, low serum AMH is suggestive of hypogonadism, reflecting a decreased Sertoli cell mass and, therefore, a reduced testicular size and/or an impaired Sertoli cell function (6, 104–112). Low AMH levels have also been suggested to predict a decreased number of germ cells in the testes (113, 114)

#### 4.2.1 Unilateral *vs* bilateral cryptorchidism

**Figure 4:**
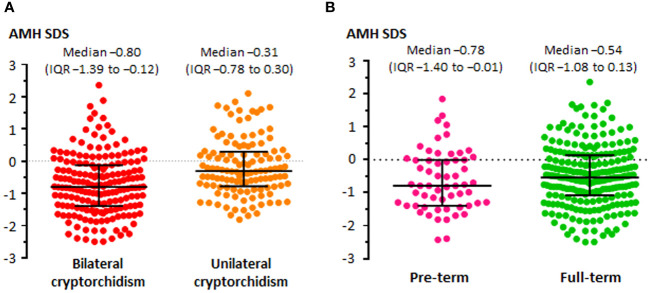
Levels of serum AMH, expressed in standard deviation scores (SDS), in boys with unilateral or bilateral cryptorchidism **(A)**, and in boys with unilateral or bilateral cryptorchidism born pre-term or full-term **(B)**. Modified with permission, from Grinspon et al. (6).

Since cryptorchidism is a sign of heterogeneous disorders (**Table 1**) (72), a wide range of serum AMH levels can be found in patients with undescended testes. As expected, testicular AMH output is more affected in boys with bilateral undescended testes than in those with unilateral cryptorchidism (**Figure 4A**). Mean or median serum AMH is significantly lower (approximately -420 to -560 pmol/l, equivalent to -60 to -80 ng/ml) in boys with bilateral cryptorchidism than in age-matched controls, while a significant difference is not always evident between boys with unilateral cryptorchidism and controls (6, 104, 113, 115). Nevertheless, a large study showed that serum AMH was clearly below the normal range in 16% of boys with unilateral cryptorchidism aged 1-6 months and in 7% of older infants and prepubertal boys. In patients with bilaterally undescended testes, serum AMH was abnormally low in 14% of infants < 6 months-old and in 19-37% of older infants and prepubertal children (6). Another study showed that serum AMH was normal in only half of the boys with cryptorchidism, without distinguishing between unilateral and bilateral forms (105). Preterm birth does not seem to have an influence on testicular function in cryptorchid boys before puberty (**Figure 4B**) (6).

#### 4.2.2 Before and after orchiopexy

**Figure 5:**
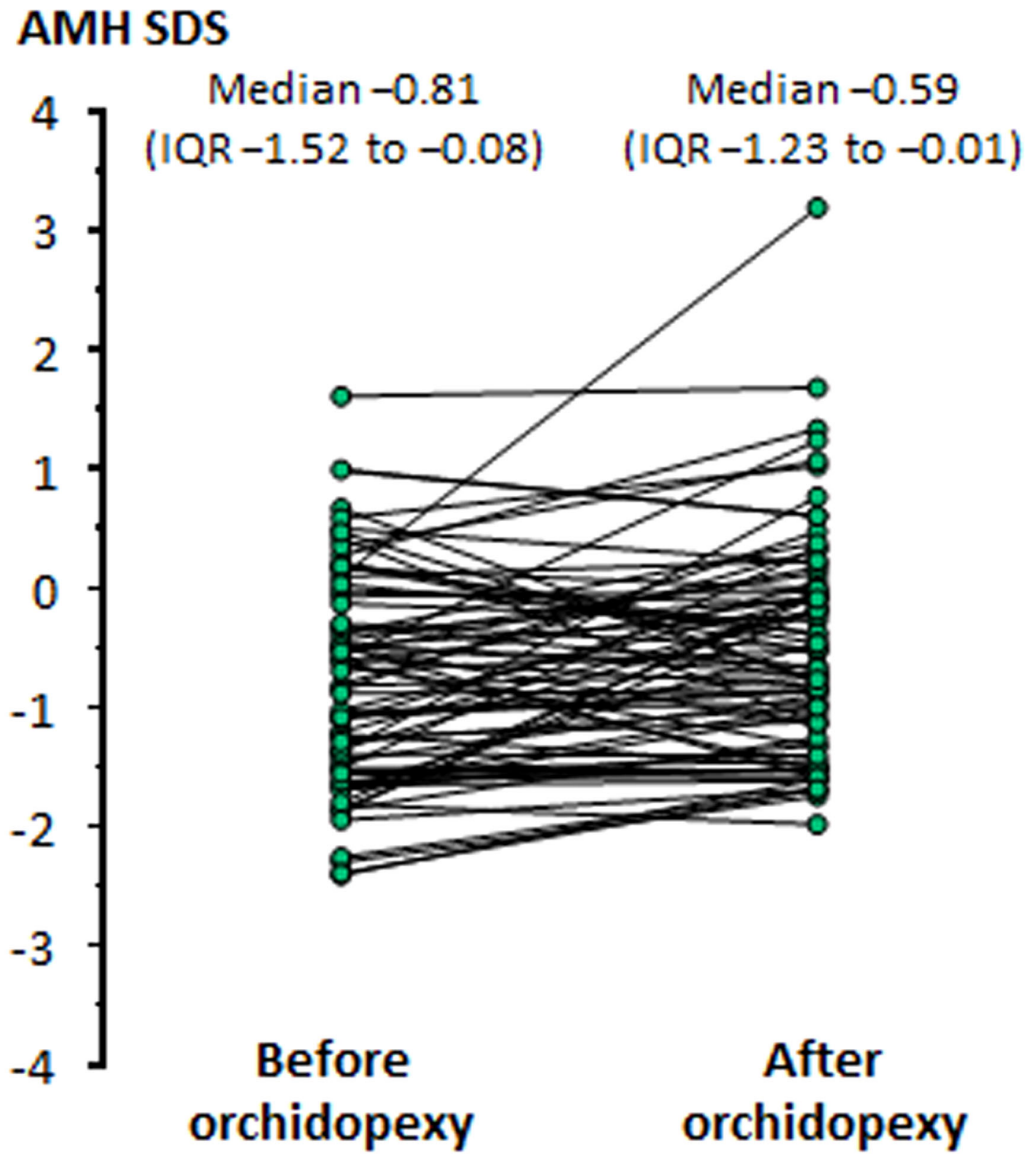
Levels of serum AMH, expressed in standard deviation scores (SDS), in boys before and after orchiopexy Modified with permission, from Grinspon et al. (6).

The impaired testicular function in patients with cryptorchidism may have two explanations: a primary gonadal dysfunction that results in a disordered testicular descent to the scrotum and, on the other hand, the longstanding stay of the gonad in an ectopic position that affects its normal function. In boys with cryptorchidism, serum AMH increases after successful orchiopexy (6, 106, 111), indicating that the correction of the abnormal position may have a positive influence, even if partial, on testicular function (**Figure 5**).

### 4.3 Assessment of testicular function in boys with cryptorchidism and associated genital anomalies

The co-occurrence of cryptorchidism with other genital anomalies, such as micropenis, hypospadias or ambiguous genitalia, or with syndromic features increases the risk of testicular dysfunction.

#### 4.3.1 Cryptorchidism and micropenis

The existence of micropenis may be a sign of defective androgen action during the second half of fetal life, which can be due to a primary testicular dysfunction such as testicular regression syndrome or to central (hypogonadotrophic) hypogonadism (116). In a newborn or infant <6 months-old with cryptorchidism and micropenis, low serum AMH associated with low testosterone and gonadotrophins are strongly suggestive of congenital central hypogonadism (108, 117). Treatment with gonadotrophins results in an increase in serum AMH and testis volume, reflecting FSH action on Sertoli cells, as well as a rise in serum testosterone, reflecting LH action on Leydig cells and leading to penile enlargement and testis descent (117–120). Testicular regression results in very low or undetectable AMH levels, together with low or undetectable levels of the other testicular hormones and elevated gonadotrophins.

#### 4.3.2 Cryptorchidism and hypospadias or ambiguous genitalia

**Figure 6:**
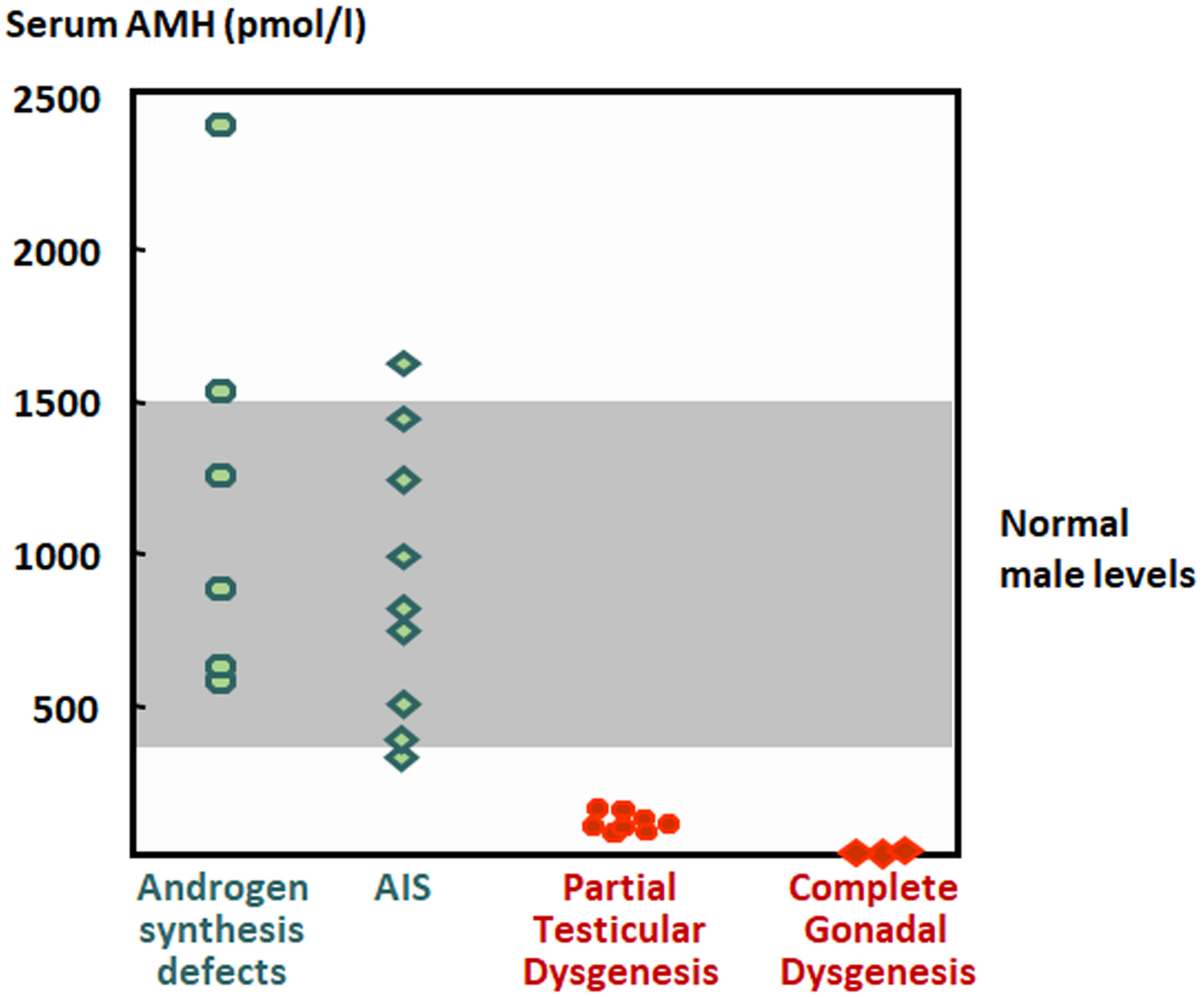
Levels of serum AMH in boys with disorders of sex development. The shaded area represents normal levels. AIS, androgen insensitivity syndrome. Modified with permission, from Rey et al. (121). ^©^ 1999 The Endocrine Society.

The coexistence of cryptorchidism with hypospadias and incompletely fused labioscrotal swellings is indicative of insufficient androgen action in the first trimester of fetal life in a 46,XY individual resulting in disorders of sex development (DSD) (70). This can be due to a disorder of gonadal development (testicular dysgenesis), or to a disorder of androgen biosynthesis (Leydig cell hypoplasia, steroidogenic enzyme defects) or action (androgen insensitivity). Serum testosterone is below the male range in both disorders of gonadal development and of androgen biosynthesis. Serum AMH determination can be useful to distinguish between them (**Figure 6**), since it is below the male range in patients with testicular dysgenesis but within the normal range or above in patients with defects of androgen synthesis (112, 121–126). The coexistence of testosterone and AMH levels in the male range is suggestive of partial androgen insensitivity (121, 124, 126).

#### 4.3.3 Cryptorchidism in genetic syndromes

Cryptorchidism is a relatively common feature of genetic syndromes resulting from sex chromosome (47,XXY Klinefelter syndrome) or autosome (Trisomy 21, Down syndrome) aneuploidies, or from single locus disorders, such as Noonan and Prader-Willi syndromes (72).

In patients with Klinefelter syndrome, serum AMH, as well as other reproductive hormones, are within the normal range in infants and children, indicating that endocrine testis function is not affected before puberty (127–130).

Trisomy 21 is characterized by an early establishment of primary hypogonadism, as reflected by lower serum AMH levels, as compared to age-matched controls, during infancy and childhood. Sertoli cell dysfunction was independent from the occurrence of cryptorchidism in patients with Down syndrome (51).

In boys with Noonan syndrome associated with *PTPN11* gene variants, serum AMH is low as compared to the general population, which suggests a Sertoli cell dysfunction; conversely, normal AMH levels have been found in boys with Noonan syndrome with pathogenic variants in *SOS1* (131).

Boys with Prader-Willi syndrome show a mild Sertoli cell dysfunction, resulting in serum AMH levels in the lower half of the reference range, with a mild increase in gonadotrophins, from early infancy (132) throughout puberty (133, 134)

## 5 Concluding remarks

AMH is a well-established biomarker of the immature Sertoli cell, from the fetal stage until mid-puberty. Its unequivocal role is to induce the regression of Müllerian ducts in the male fetus. Other physiological roles of AMH, such as its involvement in testicular descent, remain to be ascertained. Nonetheless, serum AMH determination is a useful tool to assess the existence and functional capacity of testicular tissue during infancy, childhood and early puberty in boys with cryptorchidism, either isolated or associated with micropenis or with other signs of fetal undervirilization.

## Author contributions

RR: Conceptualization, Writing – original draft, Writing – review & editing. RG: Writing – review & editing.

## Glossary

**Table:**

ACVR1	Activin receptor type 1
AMH	anti-Müllerian hormone
AMHR2	AMH receptor type 2
AP1	Activating protein 1
AP2	Activating protein 2
AR	Androgen receptor
BMP	Bone morphogenetic protein
BMPR1A	BMP receptor type 1A
cAMP	Cyclic adenosine monophosphate
DHT	Dihydrotestosterone
DSD	Disorders of sex development
ERα	Estrogen receptor α
FOXL2	Forkhead transcription factor 2
FSH	follicle stimulating hormone
GATA	Protein binding to GATA DNA sequences
GnRH	Gonadotropin-releasing hormone
GPER (GPER1 or GPR30)	G protein -coupled estrogen receptor
hCG	human chorionic gonadotropin
IκB	Inhibitor of nuclear factor kappa B
INSL3	Insulin-like peptide 3
JSRP1	Junctional sarcoplasmic reticulum 60 protein 1
LH	luteinising hormone
MAPK	Mitogen-activated protein kinase
MEK	MAPK kinase
MIF	Müllerian inhibiting factor
MIS	Müllerian inhibiting substance
Mmp2	Matrix metalloprotease 2
NFκB	Nuclear factor kappa B
Osx	Osterix
PI3K	Phosphatidylinositol 3-kinase
PKA	Protein kinase A
PKB	Protein kinase B
PMDS	Persistent Müllerian duct syndrome
PTPN11	Protein-tyrosine phosphatase nonreceptor-type 11
SF1	steroidogenic factor 1
SF3A2 (SAP62)	Splicing factor 3A 59 subunit 2
SMAD	Mothers against decapapentaplegic signalling protein
SOX9	SRY-Related HMG box gene 9
SP1	Specificity protein 1
SRY	Sex-determining region on the Y chromosome
TFII-I	General transcription factor II-I
TGFβ	Transforming factor beta
Wif1	Wnt inhibitory factor 1
WNT4	Wingless-type MMTV integration site family, member 4
